# Thioacetyl-Terminated Ferrocene-Anthraquinone Conjugates: Synthesis, Photo- and Electrochemical Properties Triggered by Protonation-Induced Intramolecular Electron Transfer

**DOI:** 10.3390/molecules15010150

**Published:** 2010-01-04

**Authors:** Wen-Wei Zhang, Mio Kondo, Takako Fujita, Kosuke Namiki, Masaki Murata, Hiroshi Nishihara

**Affiliations:** 1 Department of Chemistry, School of Science, the University of Tokyo, Hongo, Bunkyo-ku, Tokyo 113-0033, Japan; 2 State Key Laboratory of Coordination Chemistry, Coordination Chemistry Institute, School of Chemistry and Chemical Engineering, Nanjing University, Nanjing 210093, China

**Keywords:** ferrocene, anthraquinone, donor-acceptor, protonation, valence tautomerization

## Abstract

Two thioacetyl-terminated ferrocene-anthraquinone donor-acceptor molecules with different π-electron conjugative units have been synthesized via a series of Stille and Sonagashira reactions. Their photochemical and electrochemical properties before and after addition of an organic acid are investigated, indicating that these complexes are sensitive to external perturbation of protonation, leading the structural change to an expansion of π-conjugated system by cyclocondensation reaction and promoting intramolecular electron transfer from donor to acceptor. They would be good candidates for studies of novel SAMs, and the properties triggered by protonation-induced intramolecular electron transfer will make the SAMs be useful in designing new functional molecular devices.

## 1. Introduction

**Scheme 1:**
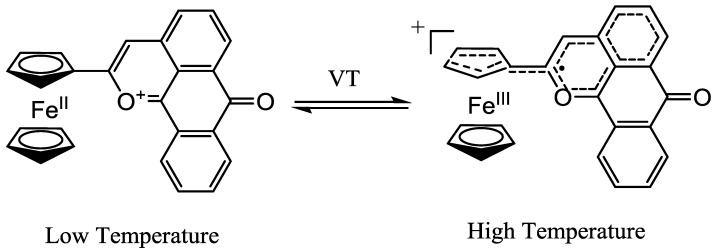
Valence tautomerism of protonated 1-FcAq in solid form.

The study of donor-acceptor (D-A) molecules is a fascinating topic, crucial for addressing fundamental aspects in academic and technological applications since a variety of biological processes and artificial devices are based on donor-acceptor interactions, such as photosynthesis and optoelectronic devices [1,2,3,4,5,6,7,8,9,10,11,12,13]. To date, a large number of donor-acceptor assemblies with photo- and electro-active units have been constructed to study their structure-property relationships for a better understanding of the electron-transfer reactions between the donor and acceptor moieties [14,15,16,17,18,19,20,21]. Recently, a π-conjugated donor-acceptor system, in which ferrocene (Fc) was used as the donor, anthraquinone (Aq) as the acceptor, and ethynylene as the π-conjugated linker, has been studied in detail in solution and solid form by our group [22,23,24,25,26,27]. It is interesting that the conjugated Fc-Aq compounds are sensitive to the external stimulus of protonation, leading to an expanded π-conjugated system formed by a cyclocondensation reaction and simultaneously displaying significant changes in their optical, structural, and magnetic properties. Moreover, the expanded π-conjugation promotes intramolecular electron transfer from donor to acceptor and results in a temperature-dependent valence tautomerization (VT) between two redox-active sites (Scheme 1) [26,27].

**Scheme 2:**
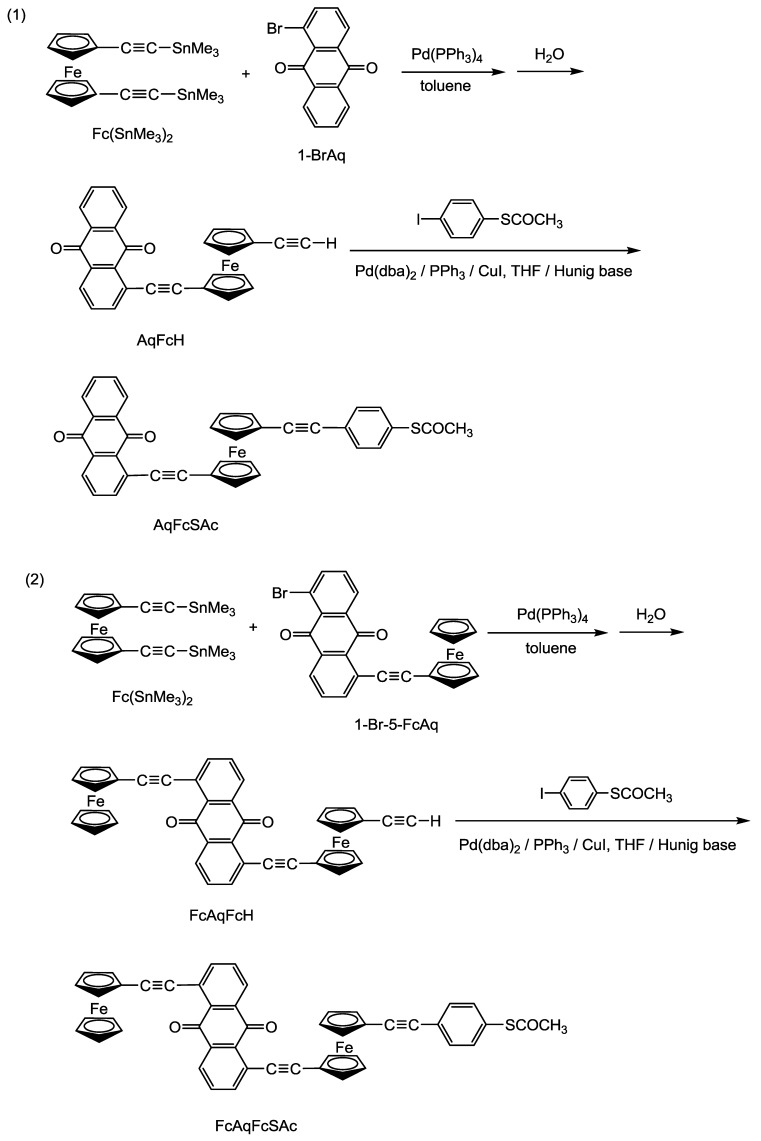
The synthesis of AqFcSAc and FcAqFcSAc.

With self-assembled monolayers (SAMs) constituting an essential element of nanotechnology and molecular engineering, this technique allows one to achieve desired functionality at a molecular scale and offers an efficient way for the engineering of nanometer-scale devices [28,29,30,31,32,33]. Nowadays SAMs capable of performing specific functions (e.g., chemical recognition, light harvesting, mechanical motion, *etc*.) are attracting much attention due to their role as promising candidates for novel classes of materials. Since the above Fc-Aq D-A complexes exhibit intriguing electroactive and switching properties in solution and the solid state, this attracted us to design complexes containing such π-conjugated systems with bistability to be immobilized on a solid support, and then to study and control their unique functions at a molecular level by physical and chemical external stimuli. Thus, two thio-derivative π-conjugated ferrocene-anthraquinone precursors capable of self-assembly on a metal substrate, abbreviated as **AqFcSAc** and **FcAqFcSAc**, were prepared (Scheme 2).

## 2. Results and Discussion

### 2.1. Synthesis

The above thio-derivative ferrocene-anthraquinone complexes were prepared by a series of Stille and Sonogashira cross-coupling reactions, as outlined in Scheme 2. **Fc(SnMe_3_)_2_** underwent Pd(PPh_3_)_4_–catalyzed Stille coupling with **1-BrAq** or **1-Br-5-FcAq**, followed by hydrolysis, to afford **AqFcH** or **FcAqFcH**. **AqFcH** or **FcAqFcH **reacted by Sonogashira coupling with 1-iodo-4-ethanethioylbenzene using Pd(dba)_2_/PPh_3_/CuI as catalysts to obtain the target compound **AqFcSAc** and **FcAqFcSAc**. It should be noted that during the Stille reaction of **Fc(SnMe_3_)_2_** with **1-BrAq** or **1-Br-5-FcAq**, the corresponding bicoupled products would be inevitably formed. Two methods could be adopted to increase the yield of the monosubstituted products of **AqFcH** and **FcAqFcH**: use of less than one-half of the equivalent amount of **1-BrAq** or **1-Br-5-FcAq** required for the reaction, and slowly dropping the solution of **1-BrAq** or **1-Br-5-FcAq** into the mixtures of **Fc(SnMe_3_)_2_** and catalysts. All the compounds were fully characterized by ^1^H-NMR, MS and EA analysis. They all showed the characteristic resonance bands of the anthraquinone moiety in the 8.3~6.9 ppm range, and the characteristic resonance bands of the ferrocene moiety in the 4.7~4.3 ppm range. As for **AqFcH** and **FcAqFcH**, they also showed the characteristic resonance bands of the ethynyl group around 2.6 ppm. While **AqFcSAc** and **FcAqFcSAc** showed the characteristic resonance bands of the thioacetyl group around 2.3 ppm. In addition, MS experiments revealed the most prominent peak of **AqFcSAc** and **FcAqFcSAc** at *m/z* 591.08 and 798.89, respectively, which were in perfect agreement with the corresponding calculated value of [M+1]^+^, and displayed an isotopic pattern identical with the simulated one. All the results given above were thus in good accordance with the proposed structures. Since other byproducts would inevitably be produced in the reactions, the yield of the corresponding products in each step was not very high.

### 2.2. Electronic properties

**Figure 1:**
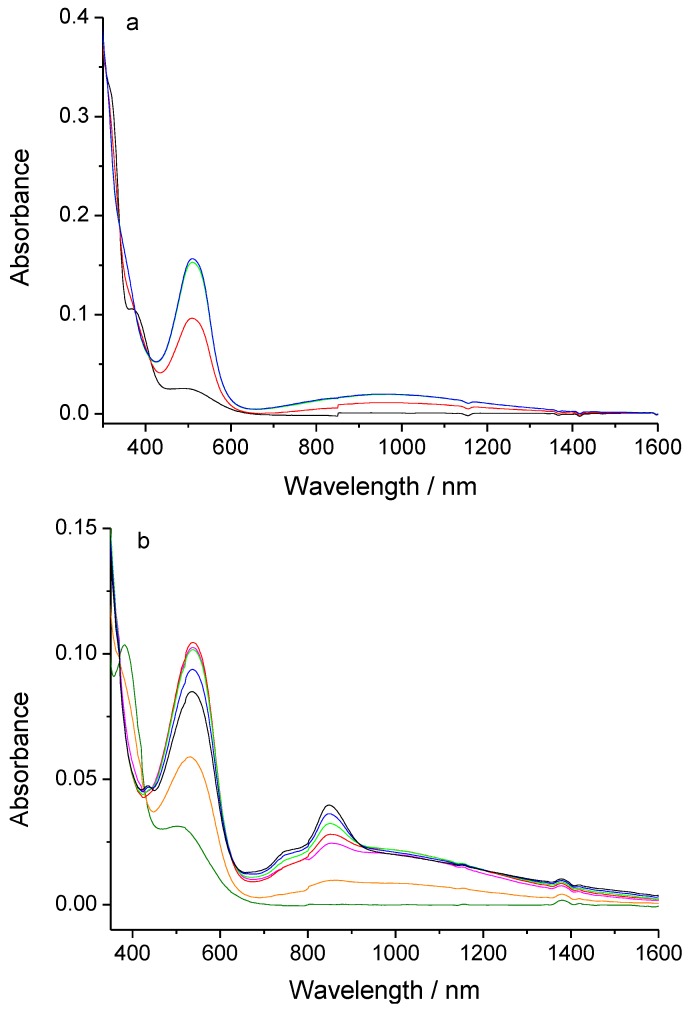
UV-vis-near-IR spectral change of 9.31 × 10^-5^ M AqFcSAc (0 (black), 0.5 (red), 1.0 (green), 2.0 (blue) equiv of TfOH) (a) and 1.15 × 10^-4^ M FcAqFcSAc (0 (olive), 0.5 (orange), 1.0 (magenta), 2.0 (red), 3.0 (green), 5.0 (blue), 8.0 (black) equiv of TfOH) (b) upon the addition of TfOH in dichloromethane.

The electronic spectra of **AqFcSAc** and **FcAqFcSAc** in dichloromethane are shown in Figure 1. The characteristic peaks of **AqFcSAc** are at 368 and 490 nm, and those of **FcAqFcSAc** are 382 and 504 nm, corresponding to n-π∗ and MLCT bands, respectively [22,23,24,25,26,27]. The introduction of the electron-withdrawing group *p*-thioacetylbenzoethynyl moiety on the ferrocene in these thio-derivative complexes led the λ_n-π__∗_ and λ_MLCT_ electronic absorbance peaks to blue shift compared with those in complexes of 1-FcAq and 1,5-Fc_2_Aq (the characteristic peaks of 1-FcAq in dichloromethane are 380 and 512 nm, and those of 1,5-Fc_2_Aq are 390 and 518 nm, respectively [22]). Similar to those of 1-FcAq and 1,5-Fc_2_Aq, the thio-derivative complexes **AqFcSAc** and **FcAqFcSAc** are also sensitive to acid, and the solution of them changed immediately from red to deep reddish-purple upon acid addition, corresponding to the UV-vis-near-IR absorption spectral change, as shown in Figure 1. We consider that cyclocondensation reaction is also performed in these two complexes upon acid addition, forming an expanded π-conjugated system [22,23,24,25,26,27].

From Figure 1, it is evident that two new bands appeared and increased in intensity at longer wavelength while the n-π∗ band of the anthraquinone moiety at lower wavelength disappeared as trifluoromethanesulfonic acid (TfOH) was stepwise added in dichloromethane solution of **AqFcSAc**. The one in the visible region at λ_max_ = 510 nm can be attributed to π -π∗ band due to the formation of an expanded π-conjugated system upon cyclocondensation, and the other broad band in the near-IR region at λ_max_ = 974 nm with the half-width Δν_1/2_ = 2.2 × 10^3^ cm^-1^ may be ascribed to the intervalence charge transfer (IVCT) transition mainly from donor to acceptor [26,27].

While for complex **FcAqFcSAc**, the case is a little different. As TfOH was gradually added, the π-π∗ band at longer wavelength (λ_max_ = 538 nm) emerged and increased in intensity below two equivalents at first, then decreased in intensity past two equivalents. Moreover, besides the new broad band corresponding to the intervalence charge transfer (IVCT) transition appeared in the near-IR region (λ_max_ = 1058 nm), two other new bands also emerged in the visible region at 738 and 852 nm, which are quite similar to those of the semiquinone form of anthraquinone derivatives [22,34].

Thus the UV-vis-near-IR spectra changes upon stepwise addition of TfOH reveal that both **AqFcSAc** and **FcAqFcSAc** respond to organic acid and undergo a protonation-induced cyclocondensation reaction, causing a structural change of the ethynylene linker to expanded π-conjugated oxygen-fused ring.

### 2.3. Electrochemical properties

**Figure 2:**
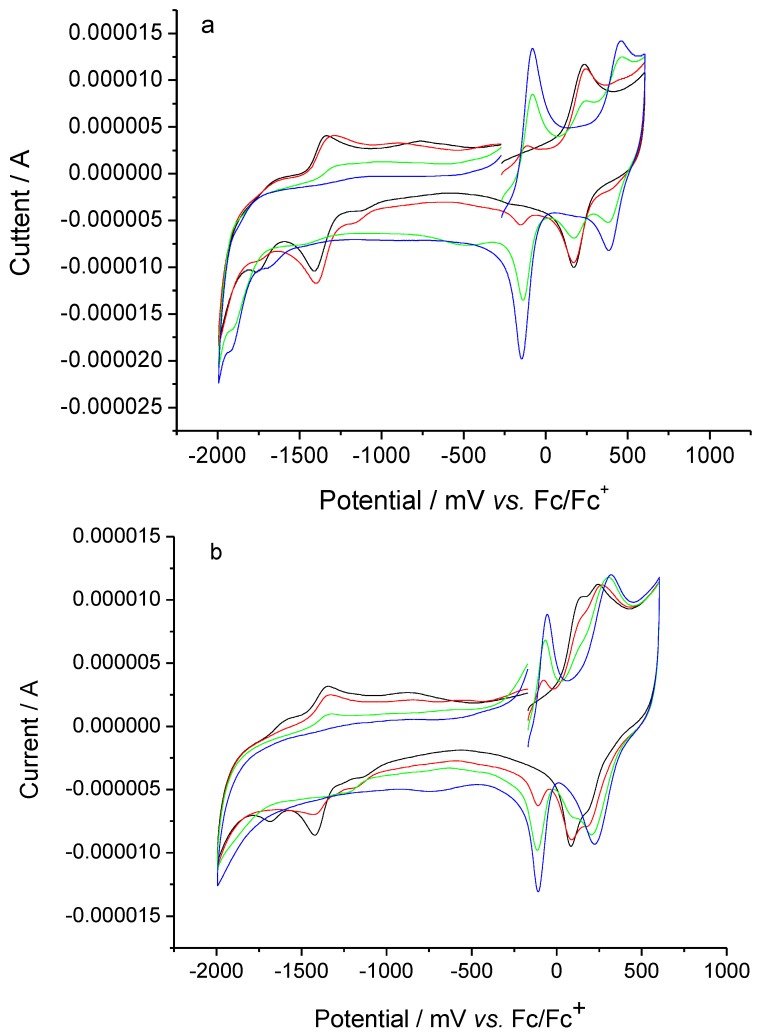
Cyclic voltammograms of 7.06 × 10^-5^ M AqFcSAc (0 (black), 0.5 (red), 1.0 (green), 2.0 (blue) equiv of TfOH) (a) and 7.51 × 10^-5^ M FcAqFcSAc (0 (black), 0.5 (red), 1.0 (green), 2.0 (blue) equiv of TfOH)(b) on addition of TfOHat GC electrode in 0.1 M n-Bu_4_NClO_4_-dichloromethane at a scan rate of 100 mV/s.

The electrochemical properties of the above thio-derivative complexes were also studied. Cyclic voltammograms of **AqFcSAc** and **FcAqFcSAc**, together with their protonated species by stepwise addition of TfOH are shown in Figure 2. The unprotonated form **AqFcSAc** exhibited a quasi-reversible (Aq^-^/Aq^2-^) and a reversible (Aq/Aq^-^) two-step 1e^-^ reduction derived from the anthraquinone moiety at ‑1,706 and ‑1,371 mV *vs*. Fc/Fc^+^ (ferrocenium/ferrocene), respectively [22,23,27,35], and a reversible 1e^-^ oxidation (Fc/Fc^+^) originated from the ferrocene moiety at 202 mV *vs*. Fc/Fc^+^ [22,23,27,36,37]. After TfOH was added in stepwise fashion, the three original redox waves gradually disappeared and two new reversible redox waves at a more positive region were observed at ‑113 mV and 419 mV *vs*. Fc/Fc^+^, respectively. The wave at -113 mV can be assigned to the reduction of the dibenzopyrylium moiety due to cyclocondensation, and the wave at 419 mV is ascribed to the oxidation of the ferrocene moiety [27].

As for **FcAqFcSAc**, it showed a quasi-reversible (Aq^-^/Aq^2^^-^) and a reversible (Aq/Aq^-^) two-step 1e^-^ reduction derived from the anthraquinone moiety at ‑1,644 and ‑1,382 mV *vs*. Fc/Fc^+^, respectively [22,23,27,35], and a little overlapping reversible two-step 1e^- ^oxidation originated from two different ferrocene moieties at 114 and 220 mV *vs*. Fc/Fc^+^, respectively. The wave around 114 mV corresponds to the ferrocene moiety not substituted by the thio-derivative group, while the wave around 220 mV corresponds to the thioacetyl benzoethynyl substituted ferrocene moiety. As the stepwise addition of TfOH, its protonated complex formed gradually, accompanied by the gradual disappearance of the redox peaks of the anthraquinone moiety instead of the formation of a new reversible wave corresponding to the dibenzopyrylium moiety at a more positive potential around ‑82 mV *vs*. Fc/Fc^+ ^[27]. Simultaneously, a new reversible redox wave at a slightly more positive potential around 270 mV *vs*. Fc/Fc^+^, corresponding to the ferrocene moiety near the dibenzopyrylium ring appeared, which overlapped with the redox peak of the ferrocene moiety adjacent to the thioacetyl benzoethynyl group. 

The most remarkable discrepancy of the electrochemical properties between the unprotonated and protonated forms is that the first reduction wave of the protonated form is largely shifted in the positive direction. The difference between the first reduction potential and the first oxidation potential of **AqFcSAc **and its protonated species is 1,573 mV and 532 mV, respectively, and that of **FcAqFcSAc **and its protonated form is 1,496 mV and 352 mV, respectively. All the above suggests that the LUMO level of the protonated formis lowered upon the cyclization reaction [27], leading to a smaller HOMO–LUMO gap and transition bands in lower energy region, which is consistent with the UV–vis–NIR results described above. And it can be seen from the data that the HOMO–LUMO energy gap of these two protonated thioacetyl-terminated ferrocene-anthraquinone complexes is smaller than that of 1-ferrocenylethynylanthraquinone (610 mV) [27], which may better promote intramolecular electron transfer in these thio-derivative protonated species. So we speculate that valence tautomerism would also exist in the solid state of the above thio-derivative protonated forms. Moreover, the electrochemical investigation reveals that the HOMO–LUMO energy gap of **FcAqFcSAc **and its protonated form is lower than that of **AqFcSAc **and its protonated form, which is in accordance with the result that the compound with the larger π-conjugated units has lower HOMO–LUMO energy gap [21]. Therefore, the electrochemical properties of the above thio-derivative complexes before and after addition of the acid also suggest cyclocondensation reaction in the presence of a strong organic acid in the Fc-Aq system.

### 2.4. SAMs on gold

The electronic and electrochemical studies indicate that these thio-derivative Fc-Aq complexes are also sensitive to external stimuli, exhibiting a protonation-induced intramolecular electron-transfer reaction in solution. In order to study this process at a molecular level and to construct functional molecular devices, an effectual way to obtain monolayers of them is by making them self-assemble on solid films. 

**Figure 3:**
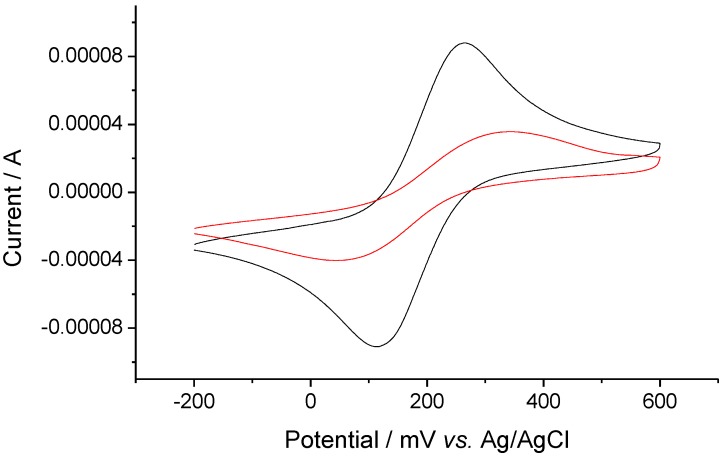
Voltammetric responses of a bare gold electrode (black) and a gold electrode modified by AqFcSAc (red) in 2 mM K_3_Fe(CN)_6_ + 0.2 M KCl at a scan rate of 100 mV/s.

By direct adsorption or by *in situ* deprotection of the thio-moieties by deacylation of thioacetyl group [38,39], **AqFcSAc** tends to be self-assembled on gold substrate, forming monolayers containing the π-conjugated donor-acceptor system, which has been demonstrated by the redox response of Fe(CN)_6_^3-/4-^ hindered by the presence of the SAMs as shown in Figure 3. But from this figure, we can see that such SAMs were less densely packed on the substrate due to the existence of defects or pinholes. To better study their particular properties at molecular level, more densely ordered SAMs on the film are necessary. Some other methods, such as coadsorption of alkyl thiols to fill-in the pinholes [40,41], or subsequent immersion into the solutions of the matrix and active species to get high-quality SAMs [42], will be under investigation.

## 3. Experimental

### 3.1. General methods

All preparations were performed under an atmosphere of dry nitrogen or dry argon, unless otherwise indicated. Solvents and reagents were used as received from commercial sources (Kanto Chemicals or Tokyo Kasei Chemicals) unless otherwise noted. Triethylamine was dried by reflux over KOH and distilled under nitrogen. Toluene and dichloromethane were distilled from CaH_2 _under nitrogen. THF was distilled from sodium and benzophenone under nitrogen. All the solvents used for the synthesis, electrochemical and spectroscopic measurements were degassed by the freeze-pump-thaw method. 

### 3.2. Spectroscopy

The ^1^H-NMR spectra of the samples in chloroform-*d_1_* or dichloromethane-*d_2_* were collected with an EX270 or ECX400 spectrometer (Bruker). Infrared absorbance spectra were recorded using a JASCO FT/IR-620V spectrometer. UV-vis-near-IR absorption spectra were acquired with a JASCO V-570 spectrometer. MALDI–TOF mass spectra were recorded with an AXIMA-CFR spectrometer (Shimadzu Kratos). Elemental analysis of the products was performed on a Yanaco MT-6 Type with C, H, N corder at the Elemental Analysis Center of the University of Tokyo.

### 3.3. Electrochemical measurements

Cyclic voltammetry was carried out in a standard one-compartment cell under an argon atmosphere equipped with a platinum-wire counter electrode and an Ag/Ag^+^ (for **AqFcSAc** and **FcAqFcSAc**) or Ag/AgCl (for SAMs on gold) reference electrode with a BAS CV-50W voltammetric analyzer. A glassy carbon working electrode of 5 mm in diameter was polished with 0.3 μm alumina, followed by an ultrasonic rinse with distilled water and acetone before each experiment. The potentials of **AqFcSAc** and **FcAqFcSAc** were corrected to the standard of Fc/Fc^+^ in CH_2_Cl_2_ (0.195 V *vs.* Ag/Ag^+^ electrode).

### 3.4. Synthesis

*1-Br-5-FcAq*: Under a N_2 _atmosphere, a solution of 1,5-dibromoanthraquinone [43] (183 mg, 0.499 mmol) in triethylamine (40 mL) was slowly added into a refluxing stirred solution of ethynylferrocene (105 mg, 0.501 mmol), bis(triphenylphosphine)palladium(II) dichloride (3.7 mg, 5.3 × 10^-3^mmol) and copper(I) iodide (1.1 mg, 5.8 × 10^-3^ mmol) dissolved in triethylamine (35 mL). The mixed solution gradually turned orange, then gave a dark brown suspension. The reaction process was monitored by TLC. After refluxing for 5 h, the solvent was evaporated under vacuum. The residue was dissolved in dichloromethane, washed with brine (3% w/w), then dried with anhydrous MgSO_4_ and filtered off. The product in the filtrate was chromatographed with an alumina column (activity: III, 6*φ* × 30 cm) using dichloromethane-hexane (1:1 v/v) as eluent. The fourth brown band was collected, and then evaporated under vacuum to give brown-red solid. The solid was recrystallized from dichloromethane-hexane (1:2, v/v) to afford needle crystals of 1-bromo-5-ferrocenylethynyl anthraquinone (**1-Br-5-FcAq**). Yield: 75.1 mg (30.4%). ^1^H-NMR (CDCl_3_): *δ* (ppm) 8.42 (dd, *J = *7.56, 1.24 Hz, 1H), 8.27 (dd, *J = *7.80, 1.44 Hz, 1H), 8.02 (dd, *J = *7.80, 1.24 Hz, 1H), 7.91 (dd, *J = *7.80, 1.48 Hz, 1H), 7.72 (t, *J = *7.80 Hz, 1H), 7.60 (t, *J = *7.80 Hz, 1H), 4.66 (t, *J = *1.96 Hz, 2H), 4.35 (s, 5H), 4.34 (t, *J = *1.92 Hz, 2H); IR (KBr): 2216 cm^-1^ (ν_C__×__C_), 1678 cm^-1^ (ν_C=O_); Anal. Calcd. for C_26_H_15_O_2_BrFe: C, 63.07 ; H, 3.05. Found: C, 62.79; H, 3.07.

*AqFcH*: Under a N_2 _atmosphere, a mixture of 1,1’-bis(trimethylstannylethynyl)ferrocene [**Fc(SnMe_3_)_2_**] [44] (261 mg, 0.466 mmol) and tetrakis(triphenylphosphine)palladium(0) [Pd(PPh_3_)_4_] (19.0 mg, 0.0164 mmol) was dissolved in toluene (25 mL) and heated to reflux, then a solution of 1-bromoanthraquinone (1-BrAq) (54.4 mg, 0.190 mmol) in toluene (15 mL) was added dropwise, during which the reactive solution was turned deep red-brown. After refluxing for 1.5 h, 10 mL water (deaerated with N_2_ for 1 h) was added, and the mixed solution was refluxed for another 0.5-1 h. After solvent removal under vacuum, the residue was dissolved in chloroform, washed with water, dried with anhydrous Na_2_SO_4_, and rotary evaporated to dryness. The desired product was isolated by column chromatography (alumina, activity: III, 2*φ* × 20 cm) using dichloromethane-hexane (1/2 v/v) as eluent. The second band was collected to afford the deep red solid **AqFcH**. Yield: 26.0 mg (31.2%). ^1^H-NMR (CDCl_3_): *δ* (ppm) 8.30 (dd, *J = *7.08, 1.72 Hz, 1H), 8.23 (m, 2H), 7.89 (dd, *J = *7.68, 1.32 Hz, 1H), 7.73 (m, 2H), 7.64 (t, *J = *7.60 Hz, 1H), 4.62 (t, *J = *1.72 Hz, 2H), 4.53 (t, *J = *1.72 Hz, 2H), 4.34 (t, *J = *1.96 Hz, 2H), 4.31 (t, *J = *1.96 Hz, 2H), 2.65 (s, 1H).

*FcAqFcH*: Under a N_2 _atmosphere, a mixture of 1,1’-bis(trimethylstannylethynyl)ferrocene (**Fc(SnMe_3_)_2_**) [44] (216 mg, 0.386 mmol) and tetrakis(triphenylphosphine)palladium(0) (Pd(PPh_3_)_4_) (46.3 mg, 0.0401 mmol) was dissolved in toluene (8 mL) and heated to reflux, then a solution of 1-bromo-5-ferrocenylethynyl anthraquinone (**1-Br-5-FcAq**) (42.7 mg, 0.0862 mmol) in toluene (8 mL) was added dropwise, and the reaction mixture gradually turned deep red-brown. After refluxing for 2 h, 10 mL water (deaerated with N_2_ for 1 h) was added, and the mixed solution was refluxed for another 0.5-1 h. Then the solvent was removed under vacuum, and the residue was dissolved in chloroform, washed with water, dried with anhydrous Na_2_SO_4_, and rotary evaporated to dryness. The desired product was isolated by column chromatography (alumina, activity: III, 2*φ* × 20 cm) using dichloromethane-hexane (1/2 v/v) as eluent. The second band was collected, and the deep red solid of **FcAqFcH** was obtained after solvent evaporation. Yield: 18.1 mg (32.4 %). ^1^H-NMR (CDCl_3_): *δ* (ppm) 8.28 (m, 2H), 7.85 (m, 2H), 7.65 (m, 2H), 4.62 (t, *J = *1.96 Hz, 2H), 4.59 (t, *J = *1.96 Hz, 2H), 4.53 (t, *J = *1.96 Hz, 2H), 4.35 (t, *J = *1.96 Hz, 2H), 4.31 (t, *J = *1.96 Hz, 2H), 4.2817 (s, 5H), 4.26 (t, *J = *1.96 Hz, 2H), 2.66 (s, 1H).

*AqFcSAc*: Under a N_2 _atmosphere, **AqFcH** (56.0 mg, 0.127 mmol), 1-iodo-4-ethanethioylbenzene [45] (44.0 mg, 0.158 mmol), bis(dibenzylideneacetone)palladium(0) (Pd(dba)_2_) (4.0 mg, 7.0 × 10^-3^ mmol), triphenylphosphine (PPh_3_) (9.0 mg, 3.4 × 10^-2^ mmol) and copper(I) iodide (CuI) (3.0 mg, 1.6 × 10^-2^ mmol) were mixed in a two-necked flask. Then diisopropylethylamine (4 mL) and freshly distilled anhydrous THF (10 mL) were added to the mixture. After stirring for 3 days, solvents were removed under vacuum. The residue was dissolved in chloroform (20 mL), washed with water (20 mL), dried with anhydrous Na_2_SO_4_, and rotary evaporated to dryness. The second brown band was collected after TLC isolation using ether-hexane (2/3 v/v) as eluent to afford the desired complex **AqFcSAc**. Yield: 19.6 mg (26.1 %). ^1^H-NMR (CD_2_Cl_2_): *δ* (ppm) 8.19 (m, 2H), 8.11 (dd, *J = *7.80, 1.34 Hz, 1H), 7.73 (m, 3H), 7.52 (t, *J = *7.80Hz, 1H), 7.16 (d, *J = *8.56 Hz, 2H), 6.97 (d, *J = *8.80 Hz, 2H), 4.62 (t, *J = *1.96Hz, 2H), 4.56 (t, *J = *1.96Hz, 2H),4.36 (m, 4H), 2.31 (s, 3H); MS: *m/z *591.08 [M+1]^+^ (calcd. 591.07); Anal. Calcd. for C_36_H_22_O_3_SFe: C, 73.23 ; H, 3.76 . Found: C, 73.67; H, 3.75.

*FcAqFcSAc*: Under a N_2 _atmosphere, **FcAqFcH** (32.7 mg, 0.0504 mmol), 1-iodo-4-ethanethioylbenzene [45] (21.6 mg, 0.0777 mmol), bis(dibenzylideneacetone)palladium(0) (Pd(dba)_2_) (3.0 mg, 5.2 × 10^-3^ mmol), triphenylphosphine (PPh_3_) (5.0 mg, 1.9 × 10^-2^ mmol) and copper(I) iodide (CuI) (3.0 mg, 1.6 × 10^-2^ mmol) were mixed in a two-necked flask. Then diisopropylethylamine (1.5 mL) and freshly distilled anhydrous THF (5 mL) were added to the mixture. After stirring for 5 days, solvents were removed under vacuum. The residue was dissolved in dichloromethane (30 mL), washed with water (20 mL), dried with anhydrous Na_2_SO_4_, and evaporated to dryness. The second brown band was collected after TLC isolation using ether-hexane (2/3 v/v) as eluent to give the desired complex **FcAqFcSAc**. Yield: 9.0 mg (22 %). ^1^H-NMR (CD_2_Cl_2_): *δ* (ppm) 8.26 (m, 2H), 7.91 (dd, *J = *7.70, 1.34 Hz, 1H), 7.79 (dd, *J = *7.70, 1.30 Hz, 1H), 7.73 (t, *J = *7.68 Hz, 1H), 7.62 (t, *J = *7.80 Hz, 1H), 7.27 (d, *J = *8.52 Hz, 2H), 7.10 (d, *J = *8.56 Hz, 2H), 4.70 (t, *J = *1.84 Hz, 2H), 4.65 (m, 4H), 4.44 (m, 4H), 4.36 (t, *J = *1.96 Hz, 2H), 4.35 (s, 5H), 2.39 (s, 3H); MS: *m/z *798.89 [M+1]^+^ (calcd. 799.07); Anal. Calcd. for C_48_H_30_O_3_SFe_2_: C, 72.20 ; H, 3.79 . Found: C, 72.58; H, 3.85.

### 3.5. SAMs preparation

SAMs for electrochemical measurements were formed on gold substrates (100 nm on mica). Prior to self-assembly, the gold substrate was annealed by hydrogen flame, then immediately immersed in a solution of **AqFcSAc** (2.2 mM) in dichloromethane or in a mixture of THF with ammonium hydroxide by *in situ* hydrolysis of thioacetyl groups to afford the free thiol (under Ar atmosphere) [40,41]. After deposition for 24 h, the SAM-covered substrate was removed from the solution and rinsed thoroughly with dichloromethane or THF, followed by drying in an Ar gas stream.

## 4. Conclusions

Two thio-derivative Fc-Aq complexes with different π-electron conjugative units capable for SAMs formation have been successfully synthesized. Their photochemical and electrochemical properties altering with external protonation demonstrated a cyclocondensation reaction in the presence of a strong organic acid. Expansion of the π-conjugated system in the protonated form results in a lowering of the LUMO orbital level and facilitates intramolecular electron transfer between donor and acceptor. More researches at a molecular level will be detailed studied in the future. 

## References

[1] Wasielewski M.R. (1992). Photoinduced Electron transfer in supramolecular systems for artificial photosynthesis. Chem. Rev..

[2] Kurreck H., Huber M. (1995). Model reactions for photosynthesis - Photoinduced charge and energy transfer between covalently linked porphyrin and quinone units. Angew. Chem. Int. Ed. Engl..

[3] Gust D., Moore T.A., Moore A.L. (2001). Mimicking photosynthetic solar energy transduction. Acc. Chem. Res..

[4] Holten D., Bocian D.F., Lindsey J.S. (2002). Probing electronic communication in covalently linked multiporphyrin arrays. A guide to the rational design of molecular photonic devices. Acc. Chem. Res.

[5] Imahori H., Mori Y., Matano Y. (2003). Nanostructured artificial photosynthesis. J. Photochem. Photobiol. C - Photo..

[6] Brabec C.J., Sariciftci N.S., Hummelen J.C. (2001). Plastic solar cells. Adv. Funct. Mater..

[7] Wienk M.W., Kroon J.M., Verhees W.J.H., Knol J., Hummelen J.C., van Hal P.A., Janssen R.A.J. (2003). Efficient methano[70]fullerene/MDMO-PPV bulk heterojunction photovoltaic cells. Angew. Chem. Int. Ed..

[8] Winder C., Sariciftci N.S. (2004). Low bandgap polymers for photon harvesting in bulk heterojunction solar cells. J. Mater. Chem..

[9] Li G., Shrotriya V., Huang J., Yao Y., Moriarty T., Emery K., Yang Y. (2005). High-efficiency solution processable polymer photovoltaic cells by self-organization of polymer blends. Nat. Mater..

[10] Ma W., Yang C., Gong X., Lee K., Heeger A.J. (2005). Thermally Stable, efficient polymer solar cells with nanoscale control of the interpenetrating network morphology. Adv. Funct. Mater..

[11] Atienza C.M., Fernández G., Sánchez L., Martín N., Dantas I.S., Wienk M.M., Janssen R.A.J., Rahman G.M.A., Guldi D.M. (2006). Light harvesting tetrafullerene nanoarray for organic solar cells. Chem. Commun..

[12] Sierra M., Herranz M.Á., Zhang S., Sánchez L., Martín N., Echegoyen L. (2006). Self-assembly of C-60 π-extended tetrathiafulvalene (exTTF) dyads on gold surfaces. Langmuir.

[13] Delgado J.L., Espíldora E., Liedtke M., Sperlich A., Rauh D., Baumann A., Deibel C., Dyakonov V., Martín N. (2009). Fullerene dimers (C60/C70) for energy harvesting. Chem. Eur. J..

[14] Okamoto K., Imahori H., Fukuzumi S. (2003). Metal ion-promoted intramolecular electron transfer in a ferrocene-naphthoquinone linked dyad. Continuous change in driving force and reorganization energy with metal ion concentration. J. Am. Chem. Soc..

[15] Thompson A.L., Ahn T.-S., Thomas K.R.J., Thayumanavan S., Martínez T.J., Bardeen C.J. (2005). Using Meta conjugation to enhance charge separation versus charge recombination in phenylacetylene donor−bridge−acceptor complexes. J. Am. Chem. Soc..

[16] Perepichka D.F., Bryce M.R. (2005). Molecules with exceptionally small HOMO-LUMO Gaps. Angew. Chem. Int. Ed..

[17] Chernick E.T., Mi Q., Kelley R.F., Weiss E.A., Jones B.A., Marks T.J., Ratner M.A., Wasielewski M.R. (2006). Electron donor-bridge-acceptor molecules with bridging nitronyl nitroxide radicals: influence of a third spin on charge- and spin-transfer dynamics. J. Am. Chem. Soc..

[18] Kim O.K., Je J., Melinger J.S. (2006). One-dimensional energy/electron transfer through a helical channel. J. Am. Chem. Soc..

[19] Murphy A.R., Frechet J.M.J. (2007). Organic semiconducting oligomers for use in thin film transistors. Chem. Rev..

[20] Kulkarni A.P., Zhu Y., Babel A., Wu P.T., Jenekhe S.A. (2008). New ambipolar organic semiconductors. 1. Synthesis, single-crystal structures, redox properties, and photophysics of phenoxazine-based donor−acceptor molecules. Chem. Mater..

[21] Zhang W.W., Mao W.L., Hu Y.X., Tian Z.Q., Wang Z.L., Meng Q.J. (2009). Phenothiazine-anthraquinone donor-acceptor molecules: Synthesis, electronic properties and DFT-TDDFT computational study. J. Phys. Chem.A.

[22] Murata M., Yamada M., Fujita T., Kojima K., Kurihara M., Kubo K., Kobayashi Y., Nishihara H. (2001). Structural conversion and spin separation in bis(ferrocenylethynyl)anthraquinones triggered by proton-coupled intramolecular electron transfer. J. Am. Chem. Soc..

[23] Murata M., Fujita T., Yamada M., Kurihara M., Nishihara H. (2000). Novel protonation-induced structural conversion of an ethynylene-bridged ferrocene-anthraquinone complex. Chem. Lett..

[24] Kondo M., Murata M., Nishihara H., Nishibori E., Aoyagi S., Yoshida M., Kinoshita Y., Sakata M. (2006). Guest-induced instant and reversible crystal-to-crystal transformation of 1,4-bis(ferrocenylethynyl)anthraquinone. Angew. Chem. Int. Ed..

[25] Kojima K., Zhang W.W.,  Kondo M., Uchikawa M., Namiki K., Fujita T., Murata M., Kobayashi Y., Nishihara H. (2007). Synthesis of p-conjugated ferrocene-anthraquinone alternating polymers and their protonation reactions. J. Inorg. Organomet. Polym. Mater..

[26] Kondo M., Uchikawa M., Zhang W.W., Namiki K., Kume S., Murata M., Kobayashi Y., Nishihara H. (2007). Protonation-induced cyclocondensation of 1-aryl ethynylanthraquinones: expanding the p conjugation. Angew. Chem. Int. Ed..

[27] Kondo M., Uchikawa M., Namiki K., Zhang W.W., Kume S., Nishibori E., Suwa H., Aoyagi S., Sakata M., Murata M., Kobayashi Y., Nishihara H. (2009). Counterion-dependent valence tautomerization of ferrocenyl-conjugated pyrylium salts. J. Am. Chem. Soc..

[28] Kramer S., Fuierer R.R., Gorman C.B. (2003). Scanning Probe lithography using self-assembled monolayers. Chem. Rev..

[29] Smith R.K., Lewis P.A., Weiss P.S. (2004). Patterning self-assembled monolayers. Prog. Surf. Sci..

[30] Love J.C., Estroff L.A., Kriebel J.K., Nuzzo R.G., Whitesides G.M. (2005). Self-assembled monolayers of thiolates on metals as a form of nanotechnology. Chem. Rev..

[31] Whitesides G.M. (2005). Nanoscience, nanotechnology, and chemistry. Small.

[32] Zotti G., Vercelli B., Berlin A. (2008). Monolayers and multilayers of conjugated polymers as nanosized electronic components. Acc. Chem. Res..

[33] Shen C., Buck M. (2009). Patterning of self-assembled monolayers based on differences in molecular conductance. Nanotechnology.

[34] Hulme B.E., Land E.J., Phillips G.O. (1972). Pulse radiolysis of 9,10-anthraquinones. J. Chem. Soc.Faraday Trans. I.

[35] Chambers J.Q., Patai S. (1974). Chemistry of Functional Groups. The Chemistry of the Quinonoid Compounds.

[36] Kubo K., Kondow H., Nishihara H. (1999). Oxidative-decomposition and electron-transfer kinetics of self-assembled monolayers of biferrocene-terminated alkanethiol on gold. Electrochemistry.

[37] Smalley J.F., Feldberg S.W., Chidsey C.E.D., Linford M.R., Newton M.D., Liu Y.P. (1995). The kinetics of electron transfer through ferrocene-terminated alkanethiol monolayers on gold. J. Phys. Chem..

[38] Tour J.M., Jones L., Pearson D.L., Lamba J.J.S., Burgin T.P., Whitesides G.M., Allara D.L., Parikh A.N., Atre S.V. (1995). Self-assembled monolayers and multilayers of conjugated thiols, a,w-dithiols, and thioacetyl-containing adsorbates. Understanding Attachments between potential molecular wires and gold surfaces. J. Am. Chem. Soc..

[39] Chen J., Wang W., Reed M.A., Rawlett A.M., Price D.W., Tour J.M. (2000). Room-temperature negative differential resistance in nanoscale molecular junctions. Appl. Phys. Lett..

[40] Bumm L.A., Arnold J.J., Cygan M.T., Dunbar T.D., Burgin T.P., Jones L., Allara D.L., Tour J.M., Weiss P.S. (1996). Are single molecular wires conducting?. Science.

[41] Cygan M.T., Dunbar T.D., Weiss P.S. (1998). Insertion, conductivity, and structures of conjugated organic oligomers in self-assembled alkanethiol monolayers on Au{111}. J. Am. Chem. Soc..

[42] Shaporenko A., Rössler K., Lang H., Zharnikov M. (2006). Self-assembled monolayers of ferrocene-substituted biphenyl ethynyl thiols on gold. J. Phys. Chem. B.

[43] Doyle M.P., Siegfried B., Dellaria J.F. (1977). Alkyl nitrite-metal halide deamination reactions. 2. Substitutive deamination of arylamines by alkyl nitrites and Copper(II) halides. A direct and remarkably efficient conversion of arylamines to aryl halides. J. Org. Chem..

[44] Long N.J., Martin A.J., Vilar R., White A.J.P., Williams D.J., Younus M. (1999). Synthesis, characterization, and theoretical studies of new alkynylferrocene and -biferrocene ligands and their platinum-containing dimers and oligomers. Organometallis.

[45] Hsung R.P., Chidsey C.E.D., Sita L.R. (1995). Synthesis and characterization of unsymmetric ferrocene-terminated phenylethynyl oligomers Cp2Fe-[C°C-C6H4]n-X (X = SH, SMe, SOMe, and SO2Me). Organometallics.

